# Supplementary figures and images for: Near-infrared transillumination imaging combined with aperture photometry for the quantification of melanin in the iris pigment epithelium
Source: PLoS One. 2020 Mar 6;15(3):e0230210. doi: 10.1371/journal.pone.0230210 (PMC7060071; doi:10.1371/journal.pone.0230210)

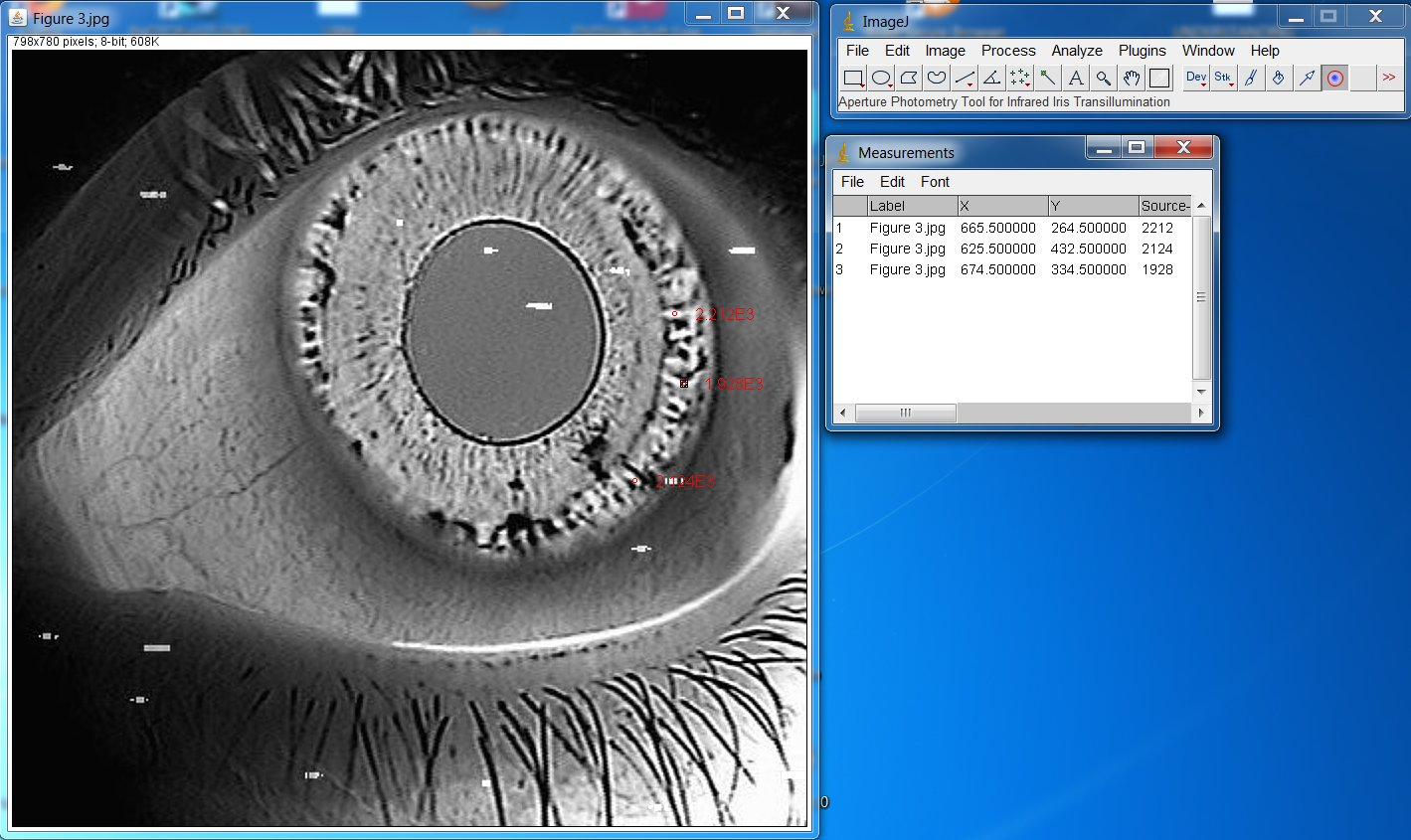

Supplement: S1 Fig — (TIF) [file pone.0230210.s001.tif]
